# A Comprehensive Review of Microfluidic Water Quality Monitoring Sensors

**DOI:** 10.3390/s19214781

**Published:** 2019-11-03

**Authors:** Swapna A. Jaywant, Khalid Mahmood Arif

**Affiliations:** Department of Mechanical and Electrical Engineering, SF&AT, Massey University, Auckland 0632, New Zealand; s.jaywant@massey.ac.nz

**Keywords:** microfluidics, water quality monitoring, heavy metals, pathogens, nutrients

## Abstract

Water crisis is a global issue due to water contamination and extremely restricted sources of fresh water. Water contamination induces severe diseases which put human lives at risk. Hence, water quality monitoring has become a prime activity worldwide. The available monitoring procedures are inadequate as most of them require expensive instrumentation, longer processing time, tedious processes, and skilled lab technicians. Therefore, a portable, sensitive, and selective sensor with in situ and continuous water quality monitoring is the current necessity. In this context, microfluidics is the promising technology to fulfill this need due to its advantages such as faster reaction times, better process control, reduced waste generation, system compactness and parallelization, reduced cost, and disposability. This paper presents a review on the latest enhancements of microfluidic-based electrochemical and optical sensors for water quality monitoring and discusses the relative merits and shortcomings of the methods.

## 1. Introduction

In this century, one of the major challenges that human beings are likely to face is water quality. Due to pollution, the amount of drinkable water is reducing day by day. This water pollution or contamination occurs due to various sources. These sources can be categorized as point sources and nonpoint sources [1,2]. Dumping of organic and inorganic wastes from industrial and domestic discards form the point sources of drinking water contaminants, whereas the nonpoint sources are land runoff, applying chemicals, or leaks from buried solid waste landfills [3]. These sources add dangerous materials such as heavy metals, nutrients, and pathogens to the surface water. Heavy metals (e.g., arsenic (As), lead (Pb), mercury (Hg), etc.) naturally exist in the surroundings, and various anthropogenic actions are also responsible for adding heavy metals to the environment [4]. Most of these heavy metals may cause fatal effects on public health due to their potentially mutagenic or carcinogenic effects on the human body [5,6,7,8]. Nutrient contamination is also a concern for water pollution. A key source of nutrients (mostly phosphorus and nitrogen) is land runoff since the nitrate and phosphorus ions are not held by soil particles. Pathogen contamination is another cause for concern [9]. Water polluted with organic waste and human and animal excrement is a potent source of pathogenic bacteria, protozoa, viruses, and parasitic worms. It results in gastrointestinal illness and can be a potential risk to human health. Escherichia coli (*E. coli*) is generally considered a faecal indicator bacteria (FIB). Bacterial counts are typically used to evaluate the influence of sewage pollution [10,11,12]. Higher concentrations of contaminants in water are responsible for fatalities across the world. The complications from water contamination are discussed in detail in the subsequent section.

The harmful outcomes of water pollution are increasing due to two main reasons: first, the level of contaminants in the water is continuously rising and there are no current practical methods to keep track of the natural changes, and the second is human growth, which leads to the need of expansion to new water sources of unknown quality. For this purpose, periodic water quality monitoring becomes very essential [13]. The traditional methods used for water monitoring are sensitive and reliable. However, mostly, they are expensive as they rely on specific instrumentation (laboratory based and not portable). Also, the samples used in these methods require transportation from the site to the laboratory, which is time consuming and, most importantly, is not field-effective. Hence, there is an increasing need to develop prompt, portable, and inexpensive sensors with high sensitivity and reliability. An ideal sensor should have a low cost, high sensitivity and selectivity, and a high throughput; should be user-friendly; and should provide in-field operation ability. It should also meet the WHO guidelines for portable sensor requirements [14]. From these perspectives, microfluidic devices are significantly appealing technologies to achieve the Lab-on-a-chip (LoC)-based point-of-care applications [15].

Microfluidics is the technology that precisely manipulates a small volume of fluids, using channels with dimensions of tens to hundreds of micrometers [16,17,18,19]. This technology has advantages such as faster reaction times, better process control, reduced waste generation, system compactness and parallelization, reduced cost, and disposability [16,20,21,22,23]. Most microfluidic devices are disposable and are used for one-time measurements. Earlier, microfluidics mainly focused on integration of microsensors with fluidic components (actuators, pumps, valves, etc.) and on miniaturization of analytical assays. Thereafter, Micro Total Analysis Systems (μTAS) evolved using micro-fabricated structures. The miniaturization with microfluidics flourished in many life science fields such as genetic analysis, cell biology, and protein analysis [24]. Currently, these devices are widely applied in all branches of science such as chemistry, biology, engineering, and biomedical sciences, etc. In earlier days, silicon was used to fabricate the microfluidic devices [25,26,27]. Then, glass and polydimethylsiloxane (PDMS) were used for fabrication purpose. Nowadays, even thermoplastic and paper are accepted as fabrication materials [15,28,29]. Several manufacturing techniques are available for microfluidic sensors such as injection moulding, softlithography, and mass-production technologies like etching. Among these methods, the softlithography technique using polydimethylsiloxane (PDMS) is a highly popular method [30,31]. However, this process requires special equipment and, in many cases, access to a clean room [32]. Currently, researchers are also making use of commercial 3-D printers to fabricate microfluidic sensors as it is possible to fabricate the microstructures in one step from a computer-based design. The frequently used approaches are inkjet printing, stereolithography (SLA), extrusion printing, etc. [33,34].

**Figure 1:**
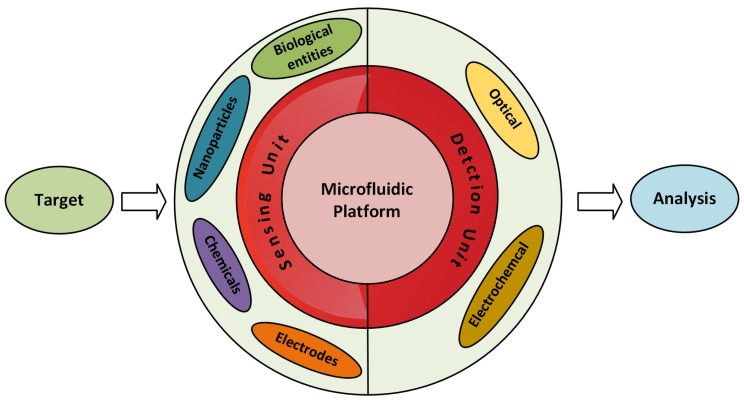
Illustration of a microfluidic sensing system.

Microfluidic sensors can be categorised in two types: one in which the microfluidic system measures the parameters inside it and the other in which the measurement of parameters takes place with the help of external integrated equipment [35,36,37]. Figure 1 represents the microfluidic system in two main parts: the sensing unit and the detection unit. The sensing unit involves elements such as biological entities, functionalized nanoparticles, and metal electrodes, etc., whereas the most commonly used detection systems with microfluidics are optical- and electrochemical-based systems [36,38]. It is possible to perform multiple analyses on the microfluidic platform by just modifying its microchannel patterns. Micromixers have a pivotal point in enhancing the sensitivity of the microfluidic-based sensors [32,39,40]. Any extensive pre-analysis is not necessary while detecting the pollutants using microfluidic sensors. Hence, microfluidic LoC devices have been broadly studied as a substitute for the conventional lab-based methods. Recent reviews presented contamination related to heavy metal [41], nutrients [42], and pathogens [43,44] individually.

This paper describes the current developments in microfluidic sensors for overall water quality monitoring that includes heavy metals, nutrients, and pathogenic detection. Specific emphasis is given on the role of microstructures in sensors. Methods outlined here are categorized on the basis of the transduction system including electrochemical and optical detection. Electrochemical detection covers techniques like electrochemical impedance spectroscopy (EIS), cyclic voltammetry (CV), and square-wave anodic stripping voltammetry (SWASV), etc. On the other hand, optical detection covers colorimetric, fluorescent, chemiluminescence (CL), surface-enhanced Raman scattering (SERS), and surface plasmon resonance (SPR) sensors. The advantages and limitations of each method along with their challenges while implementing field-effective sensors are also discussed in this review.

## 2. The Consequences of Water Pollutants on the Human Body

Water pollution is a widespread problem across the world. It impacts human life in all its aspects including mental, social, economic, physical, and emotional development. Diseases caused by contaminated drinking water result in the death of a million people every year—the majority of which are children [45]. The basic water pollutants are chemical and biological elements. Chemical pollutants include nutrients and organic and inorganic constituents, whereas biological contaminants include pathogens. Inorganic constituents include heavy metals [46]. Table 1 summarizes some effects among populations exposed to these impurities [47,48] and also includes the maximum permissible limit of the contaminant along with contamination sources [49,50,51,52,53].

Environmental vulnerability resulting from heavy metals is gaining awareness worldwide due to extensive pollution in different parts of the world [8]. Heavy metals are commonly present in most surroundings. Their physiological and chemical characteristics make them extensively useful in various industrial fields. Industrial waste enhances the possibility of heavy metal exposure, which leads to environmental pollution [83], such as for surface water, and to soil contamination. Almost all heavy metals contain toxic substances. The presence of heavy metal ions like cadmium, arsenic, chromium, lead, and mercury in water produce harmful long-term effects on human health [5,84]. Arsenic (As) exists in both the organic and inorganic form in nature, and it has different types [85]. Among all the types, As(III) and As(V) are present abundantly in natural water and are highly toxic [86,87,88]. Arsenic exposure occurs through air, food, and water [89]. Long-term inorganic arsenic toxicity can affect the cardiovascular, nervous, endocrine, and renal systems. It leads to skin lesions, pulmonary disease, hypertension, etc. [54]. Furthermore, arsenic toxicity causes different types of cancers [55,56,57,59]. Cadmium (Cd) is a malleable silver-white toxic metal that appears in the earth’s outermost layer. Its pollution naturally occurs due to volcanic eruptions, weathering, and river transport. Man-made activities like mining, smelting, tobacco smoking, disposal of sewage, etc. are equally responsible for pollution [65]. The International Agency for Research on Cancer has classified Cd and its compounds as Group 1 carcinogens. Cd toxicity causes osteoporosis, renal dysfunction, preterm birth, and low birth weights [58,66,67]. Chromium (Cr) is a steely-grey shiny metal that naturally is present in rocks, soil, animals, and plants. Industrial sources such as magnetic tapes, metal alloys, protective metal coatings, paint pigments, paper, rubber, and cement, etc. release Cr in the environment [68]. Low-level Cr toxicity can cause types of ulcers and low blood sugar. Severe chromium toxicity can develop into lung cancer, gastrointestinal cancer, and DNA damage [47,58,69]. Lead (Pb) is a shiny bluish-gray soft metal naturally present in the earth’s crust. However, mostly, it is accumulated in the environment due to activities like manufacturing, mining, and fossil fuel burning [61]. The Environmental Protection Agency (EPA) has considered Pb to be a carcinogen. Acute or short-term exposure to Pb may result in appetite loss, loss of hunger, headache, elevated blood pressure, stomachache, kidney dysfunction, exhaustion, insomnia, painful inflammation and stiffness of the joints, and vertigo. Chronic or long-term exposure to Pb can cause mental abnormality, congenital disorder, allergies, weight loss, paralysis, weak muscles, dementia, and renal damage and may even be fatal [58,60]. Mercury (Hg) is a silvery liquid metal. Its contamination occurs in its surroundings due to industrial activities like paper and pulp preservatives, pharmaceuticals, cement production, and agriculture industry, etc. [64]. Increased levels of metallic, organic, and inorganic mercury can lead to impairment of the brain, kidneys, muscles, and the fetus. It causes hypertension, cardiovascular consequences (coronary heart disease, myocardial infarction, cardiac arrhythmia, etc.), and sudden death [63]. EPA has reported methyl mercury and mercuric chloride as extremely carcinogenic compounds [58,62].

Nutrients like nitrogen and phosphorous are significant contributors to water body pollution. Abundant nitrogen occurs naturally in our surroundings as approximately 80% of the air is comprised of nitrogen. When this atmospheric nitrogen encounters rainwater, it produces nitrate and ammonium [90,91]. Further, a reduction of nitrate results in nitrite ions [74,92]. These ions can enter into the soil or surface water. The excessive use of fertilizers in agriculture is the principal nonpoint source of nitrogen and phosphorus. Another source of agricultural pollution is animal dung. In addition to this, the disposal of industrial waste and sewage is a significant anthropogenic source of nitrate pollution [7,93]. The excessive presence of nitrite and nitrate ions causes adverse health effects [76,77,78,79].

Nitrate is an essential ion for the human body to decrease blood pressure and to improve blood flow. Still, its unnecessary intake can affect the human body. It can develop diseases like gastric cancer and Parkinson’s disease. Newborns can be afflicted with blue baby syndrome [74]. It also provides a risk of thyroid cancer [75]. In rivers or lakes, the presence of excessive nitrate produces unnecessary algae and phytoplankton, which causes eutrophication. This unwanted growth of algae and phytoplankton absorbs more marine oxygen through the decomposition process and badly affects aquatic life [90].

Domestic wastewater handling and disposal methods provoke the pathogenic contamination of water bodies. Pathogenic contamination results in developing viruses, bacteria, and protozoa in water [82]. Bacteria like *Escherichia coli*, Enterococci, Bacteroides, etc. are present in the intestines of warm-blooded animals. These are recognized as indicators of faecal pollution [94,95]. These bacteria can enter into the ground water due to sewage leakage from septic tanks [80] and are responsible for waterborne diseases such as severe cholera, diarrhea, legionellosis, and typhoid fever [81]. Additionally, in the contaminated water supply, rotaviruses, hepatitis A and E viruses, and the parasitic protozoa Giardia lamblia are frequently observed [46]. Monitoring of pathogenic pollution is also equally important as many outbreaks of *E. coli* have been reported worldwide to result in infections and deaths [96,97].

## 3. Microfluidic with Electrochemical Detection

Generally, the conventional electrochemical methods include a three-electrode system containing a reference electrode, a working electrode, and a counter electrode. An interaction between the analyte and electrode surface produces an electrical signal. According to this working principle, the detection method can be classified as amperometric, voltammetric, and potentiometric [98]. Measuring micro-volumes of the sample was difficult with the silver (Ag) electrode-based methods though it has a high sensitivity towards heavy metal detection [99]. The majority of these methods needed equipment like a rotator, stirrer, etc. Such limitations have been eliminated with the help of microfabrication technologies by incorporating them on the microfluidic platform. The reference, measuring, and working electrode can be included in a microfluidic channel [100]. This miniaturization provides many advantages such as higher processing speed, mass production, portability, reduced cost, multiple analysis, and simplicity [41]. These microfluidic electrochemical sensors can be used in point-of-care (POC) applications for water quality monitoring. For the last decade, microfluidic-based electrochemical sensors have been the subject of considerable study. Several research based sensors are discussed and listed in Table 2, and commercially available sensors are listed in Table 3.

### 3.1. Heavy Metal Detection

**Figure 2:**
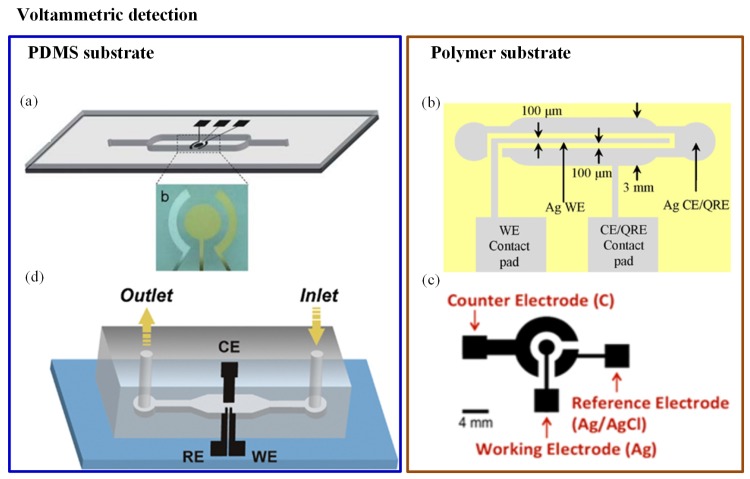
Different orientations of electrodes in electrochemical detection methods: (**a**) Schematic of an Au–Ag–Au electrode integrated with a microfluidic channel to detect Hg^+2^ [101]; (**b**) illustration of a reusable polymer chip for detection of Pb^+2^ [99]; (**c**) electrodes printed on a plastic substrate to detect As(III) [102]; and (**d**) single-walled carbon nanotube (SWCNT) electrodes for As(III) detection [103].

Chen et al. [101] developed a Hg^+2^ detector with high sensitivity and reproducibility. A three-electrode system (Au–Ag–Au) was integrated with a microfluidic channel, as illustrated in Figure 2a. A novel microfabrication technology (two-step photolithography) was used to develop the sensor. It turned out to be a disposable sensor due to reduced cost and less reactant consumption. Anodic stripping voltametry and differential pulse voltametry electrochemical analysis were used for detecting the Hg^+2^ ions. The low detection limit (3 ppb) was achieved by this sensor [101]. A similar three-electrode-based reusable polymer lab chip sensor was developed by Jung et al. [99] for Pb^+2^ detection, as shown in Figure 2b. A SWASV was used to perform Pb^+2^ analysis; the sensor was highly sensitive and environmentally friendly. The achieved limit of detection (LOD) was 0.55 ppb with 300-second deposition time. Another three-electrode system was used in As detection, as shown in Figure 2c. In this system, a disposable plastic substrate was used to print the electrodes (carbon, silver, and silver/silver chloride ink). When a water drop was introduced at the electrodes, the induced current was measured with the help of CV. The method could detect As with LOD of 1 ppb [102]. One more unique method was reported in which the integration of gold nanoparticles with a microfluidic channel was performed using electrochemical deposition. It consisted of three electrodes, gold nanoparticles, and a microfluidic channel. Electrodes were constructed with single-walled carbon nanotubes (SWCNTs) and placed into a microfluidic device as shown in Figure 2d. Gold nanoparticles were used as an electrolyte material for glucose and arsenic detection. SWASV measurements were done for ultratrace As(III) analysis. The device provided rapid and sensitive results; it could detect up to 4.5 ppb within 60 s [103]. In 2016, a electrochemical sensor was screen-printed on flexible polyethylene terephthalate material. It was demonstrated for selective monitoring of Pb^+2^ and Hg^+2^ metal ions, where electrodes were metalized by carbon- and silver-based inks. The results were reported using CV. The average peak current’s shift was noticed at 50 μM of Hg^+2^ and Pb^+2^. However, the system was not portable as it did not consist of a readout circuit [104].

**Figure 3:**
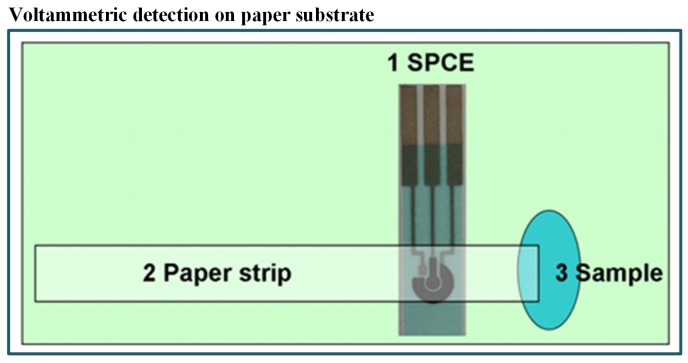
Schematic of paper-based method including integrated commercial screen-printed carbon electrodes with filter paper strips for detection of Pb^+2^ and Cd^+2^ [111].

In the last few years, paper-based microfluidics has became popular due to the following benefits: first, no need of components like pumps and tubes as it works on capillary forces and, secondly, its cost-effectiveness [105,106,107,108,109,110]. As an example, an economical and simple microfluidic paper-based electrochemical sensing device (μPED) has been fabricated by Shi et al. [111] for detecting Pb^+2^ and Cd^+2^ in aqueous samples. They have integrated commercial screen-printed carbon electrodes with filter paper strips as shown in Figure 3. The electrochemical technique was also linked with biological engineering. In such a combined system, the signal produced by a biosensor is analyzed through a three-electrode system. The detection was carried out with the help of SWASV and found a very good limit of detection (2.0 ppb for Pb^+2^ and 2.3 ppb for Cd^+2^). The device also exhibited high sensitivity and stability with real samples without pretreatment of the water sample. Some researchers have also recommended the use of bioreporters for heavy metal detection. For example, Cortés-Salazar et al. [112] utilized the natural *E. coli* defence system against toxic As(III).

**Figure 4:**
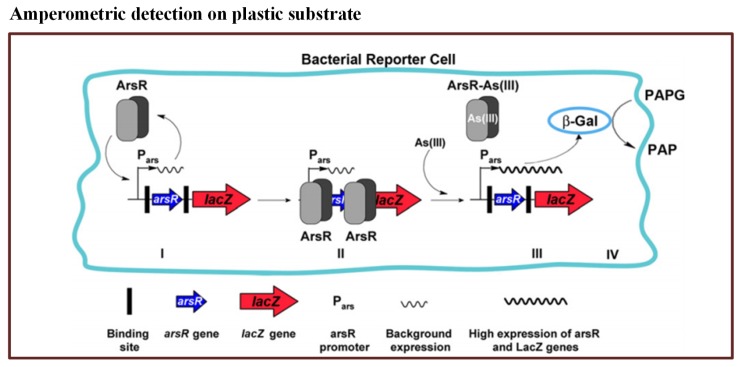
Pictorial presentation of the working scheme of the As(III) bioreporter [112].

In this method, they used a commercially available disposable microchip. It contained 16 independent electrochemical cells. The *E. coli* reporter strain was filled in the microchip. When the bioreporter encountered arsenic, β-Gal activity was produced within 25 min–50 min. The reported LOD for the method was 0.8 ppb. The principle of bioreporter is illustrated in Figure 4. Thus, this microfluidic biosensor has potential to detect arsenic with high sensitivity.

### 3.2. Nutrients

**Figure 5:**
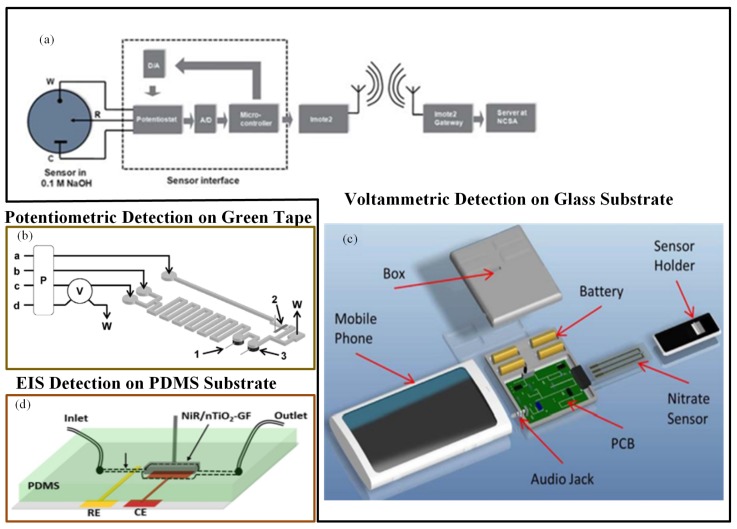
(**a**) Nitrate sensor chip with wireless communication interface [113]; (**b**) experimental set up of low-temperature co-fired ceramics (LTCC)-based continuous flow potentiometric microanalyzer to determine potassium and nitrate [115]; (**c**) a mobile sensing platform with a plug-n-play microelectronic ionic sensor to detect nitrate [114]; and (**d**) nTiO2-modified graphene foam (GF)-based nitrate sensor [117].

Gartia et al. [113] fabricated an economical, sensitive, and portable electrochemical-based measurement system for quantitative detection of nitrate in a groundwater sample (Figure 5a). The sensor chip was fabricated on a glass substrate. The working and reference electrodes were made up of a thin layer of silver. The counter electrode was a gold-deposited thin layer. The uniformity of current distribution between electrodes was enhanced using a concentric layout for the counter and working electrodes. They fabricated a miniaturized potentiostat circuit with wireless interface to make the sensor field-deployable. When the performances of a microsensor and a macroelectrode-based electrochemical system were compared, the precise examination proved that the convention macroelectrodes had far less sensitivity than the microsensor. The CV determination of nitrate ions in numerous water specimens was performed using the sensor. The LOD for the microsensor was approximately 25 ppb.

In a similar manner, Wang et al. [114] developed a mobile phone electrochemical sensing platform for nitrate quantification, as shown in Figure 5c. A mobile phone sensing platform included a plug-n-play microelectronic ionic sensor, which performed electrochemical computation utilizing the smartphone audio jack. A LOC sensing system incorporated a microelectrochemical sensor, a mobile app, and a controlling unit to control the microfluidics along with the sensor and to manage the liquid specimens. On the glass substrate, reference and working electrodes were constructed from a silver layer, and gold layer was used to create the counter electrode. The assay utilized an audio jack to interface the sensor instead of a camera. A user-friendly mobile application interface made the testing procedure very simple to use. This compact smartphone-based application could determine nitrate concentration with a LOD of 0.2 ppm in 60 s. Additionally, the mobile app could save the data on cloud servers.

**Figure 6:**
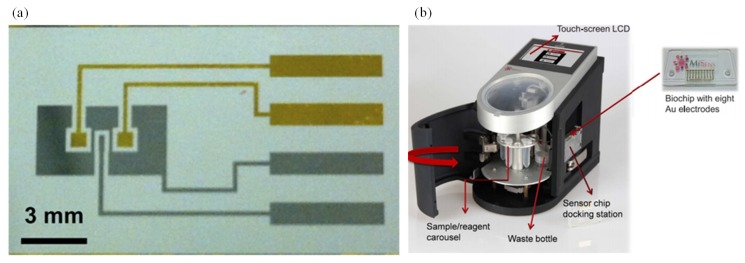
(**a**) Electrochemical DNA-based sensor for *E. coli* determination [118] and (**b**) custom-made automatic biosensor for pathogenic detection [120].

Calvo-López et al. [115] developed a compact low-temperature co-fired ceramics (LTCC)-based continuous flow potentiometric microanalyzer prototype to concurrently detect the occurrence of nitrate and potassium ions in the specimens of water recycling process (Figure 6b). The microsystem combined microfluidics with the sensing mechanism within the same substrate. The detection system comprised of two ion-selective electrodes that were constructed using a screen-printed Ag/AgCl reference electrode and ion-selective membranes. Detection limits were 0.81 mg/L and 9.56 mg/L for potassium and nitrate ions, respectively.

Generally, voltammetric procedures are easy, quick, and inexpensive and do not require specimen pretreatment before the investigation of the ions in the real specimens. Still, the production of electrodes that are modified chemically is the main obstruction in such sensors. Enhancing the ability to transfer electrons between the electrode surfaces and the electroactive analytes is the principal objective of modified electrodes. Many carbon nanostructured materials like multiwall carbon nanotubes (MWCNTs), graphene, and metal nanoparticles have been adopted extensively for accomplishing this purpose [133]. Cuartero et al. [116] reported the use of such carbon nanotubes in their method. They developed a technique to determine nitrate in seawater using the direct potentiometric method by in-line coupling to an electrochemical desalination module. Generally, the presence of highly concentrated sodium chloride in seawater causes difficulties in determining nutrient nitrite, dihydrogen phosphate, and nitrate at low micromolar levels. In traditional analytical procedures like colorimetry, UV absorption, fluorescence, chemiluminescence, and ion chromatography applied for estimating nitrate levels in seawater, very complex pretreatment is necessary. In this method, a different strategy was accomplished for the reduction of chloride concentration with a simple electrochemical transformation. A custom-made microfluidic-based flat desalination cell was combined with the potentiometric sensor (flow cell). The flow cell included an ion-to-electron transducer and a miniaturized reference electrode, where the transducer was made of lipophilic carbon nanotube (f-MWCNT)-based nitrate-selective electrode. The LOD of this assay was 5×10^−7^ M. Bagheri et al. [77] fabricated a novel method in which they deposited CuNPs upon MWCNT-reduced graphene oxide nanosheets (Cu/MWCNT/RGO) and detected nitrite and nitrate ions individually and simultaneously. The sensitivity and selectivity of GCE was improved due to the nanoparticles deposition on the MWCNT-RGO nanocomposite. The output recorded the concentrations of both ions within a span of 0.1 to 75 μM while determining the analyte simultaneously. The LOD for nitrite ion was 30 nM and for nitrate ion was 20 nM.

Ali et al. [117] described a microfluidic sensor in which nitrate monitoring was performed with the help of the EIS technique. The electrochemical electrode used in the method was a porous graphene foam (GF) scaffold. Electrochemical response was improved by modifying the GF scaffold with electrospun nTiO2, and nitrate selectivity was increased by modifying the scaffold with nitrate reductase (NiR) enzyme molecules. Nitrate solutions passed over the nTiO2-activated porous GF, and very good interaction with distinct receptor NiR bound at the scaffold surfaces occurred for nitrate detection (Figure 5d). The sensor had high sensitivity and selectivity and a rapid detection time in nitrate ion quantification.

### 3.3. Pathogens

Pathogen detection can be performed with a DNA/protein/cell-based probe. Nucleic acid detection has been recognized as a highly sensitive and selective technology. DNA-based pathogen analysis can be obtained either by direct target probing or after target amplification. Kim et al. [118] designed a compact, low-cost, electrochemical DNA-based sensor to provide real-time, continuous monitoring of pathogens. A mobile interface was coupled with the sensor that provided the analysis in terms of safe or unsafe water. The electrochemical sensor consisted of two working electrodes with platinum-based reference and counter electrode (Figure 6a). Immobilization of the working electrode was done with a DNA probe in the stem-loop structure. The methylene blue (MB) provided the electron transfer, which resulted in the current peak. When *E. coli* was introduced into the chamber, hybridization of the DNA probe took place. This resulted in the opening of the stem-loop structure which further resulted in a reduction of the current peak. This method provided qualitative results and was suitable for POC use. Li et al. [119] fabricated another electrochemical DNA-based sensor to detect hepatitis B virus (HBV). It was a simple paper-based biosensor designed with an origami paper structure and was functionalized with a DNA-modified AgNP. The use of DNA increased the speed, stability, and robustness of the biosensor. Its LOD was 85 pM.

Altintas et al. [120] fabricated a custom-made fully automatic biosensor for pathogen quantification. This device involved a novel biochip design integrated with the microfluidic system along with real-time amperometric measurements. The microfluidic system consisted of a plug-and-play-type biochip docking station that also served as a flow cell for the electrode array along with the electronic connections (Figure 6b). The sensor surface was modified with the self-assembled monolayer (SAM) of mercaptoundecanoic acid and placed. SAM-coated electrode arrays were then activated with polyclonal rabbit anti-*E. Coli* antibody. Then, an *E. coli* sample was introduced on the electrode surface. Subsequently, a horse radish peroxidase-coupled detector antibody was injected. Thus, the sandwich immunoassay was used for determination of *E. coli*. This work reported a rapid, sensitive, and specific detection of a waterborne pathogen *E. coli*. The sensor output was enhanced through the use of gold nanoparticles when compared with the standard sandwich method. The detection limit was 50 colony forming units (CFU)/mL.

The EIS method can illustrate various characteristics of electrochemical technique such as adsorption, capacitance, diffusion coefficients, electron transfer rate constants, and charge transfer resistances. Its cost-effectiveness, simplicity, and sensitivity have allowed researchers in the recent past to use it in a bio-sensing platform with many label-free transduction methods including impedance flow cytometers and Coulter counters [134,135,136].

A sample of particles scattered in a liquid is guided in the direction of electrodes through a microfluidic channel when an alternating electric field is applied in EIS. The size and configuration of the particles are responsible for alterations in electric field to particle displacements. Electrical current analysis is used to measure these alterations [136]. Several such examples of EIS-based detection methods are reviewed in the following paragraph.

Kim et al. [121] reported a label-free *E. coli* detection method that utilized positive dielectrophoretic (pDEP) focusing, capturing, and impedance measurement. This (pDEP)-based system consisted of an *E. coli*-focusing and -sensing electrode. Inclusion of the passivation layer avoided the adhesion of *E. coli* to the electrode. The change in impedance occurred due to trapping of the *E. coli* cell on the sensor electrode. The assay evaluated 300 CFU/mL within 1 min. Jiang et al. [122] designed a portable microfluidic smartphone-based EIS sensor with Bluetooth connectivity. The microfluidic sensor consisted of a microhole array silicon substrate with interdigitated sensing electrodes on it and a sensing microfluidic chamber aligned with a nano-porous filter paper. This filter paper allowed bacteria to pass through while blocking big dirt particles in water samples. The unit also included an impedance network analyzer chip with a microcontroller to perform EIS measurement and analysis. The developed android-based software app was able to remotely control the microcontroller through Bluetooth. The app could perform functions like a commercially available LCR meter. The LOD for the bacteria sensing was 10 *E. coli* cells per milliliter. Clausen et al. [123] developed another impedance-based real time microfluidic sensor to measure the bacteria levels in water samples with the water samples flowing continuously through the sensor. This method could discriminate *E. coli* from solid particles with the help of an electrical response in the high-frequency phase. Additionally, the method was able to recognize different bacteria cells: *Staphylococcus aureus* (*S. aureus*) and *E. coli*. It provided LOD of 522 cells/mL with real-time continuous monitoring of bacteria in aqueous sample utilizing impedance flow cytometry. Maw et al. [124] utilized a submicron-resistive pulse sensor based on the Coulter principle for *E. coli* monitoring. The sensitivity of this method was improved due to sample handling in a microfluidic chip and the phenomena of microscale hydrodynamic flow. The unit was made up of a supply section, base unit, detection system, data acquisition system, signal processing unit, and display unit. The base unit comprised of a PDMS microfluidic chip and four electrodes, and detection occurred at the microfluidic platform. This label-free and automatic method provided a rapid result and appeared to be a user-friendly device.

Ghosh et al. [125] presented an economical and easy microfluidic biosensor for quick and accurate measurement of salmonella typhimurium. The microfluidic chip involved the interdigitated electrode array. The electrode array surface was immobilized with anti-salmonella antibodies. The biosensor provided qualitative as well as quantitative impedance analyses within 3 h. Its LOD was $3\times 10^{3}$ CFU/mL. The authors also compared the performance of the microfluidic biosensor with the non-microfluidic biosensor. They found two to three times higher impedance response for the microfluidic biosensor with lower LOD compared to the non-microfluidic biosensor.

## 4. Microfluidic with Optical Detection

Many electrochemical methods have been presented for water quality monitoring in this review paper. The optical strategies due to their simplicity and cost-effectiveness are equally popular too. In optical-based microfluidic devices, the optical changes happen due to the chelation between the recognition element and the target constituents. These optical-based microfluidic devices are based on various techniques such as colorimerty, CL, fluorescence, SERS, and SPR. The colorimetric devices include measurement of the colour change associated with the reaction between the analyte and the sensing element. The colour variation can be observed by eye or with the help of an optical detection method [137]. In the fluorescence detection method, the analyte-induced changes are responsible for variations in characteristics of fluorochromes including fluorescence intensity, fluorescence polarization, and lifetime [41]. Several research-based examples of such detection methods are considered in the following paragraphs and are described in Table 4.

Some commercially available sensors are listed in Table 5.

### 4.1. Heavy Metal

**Figure 7:**
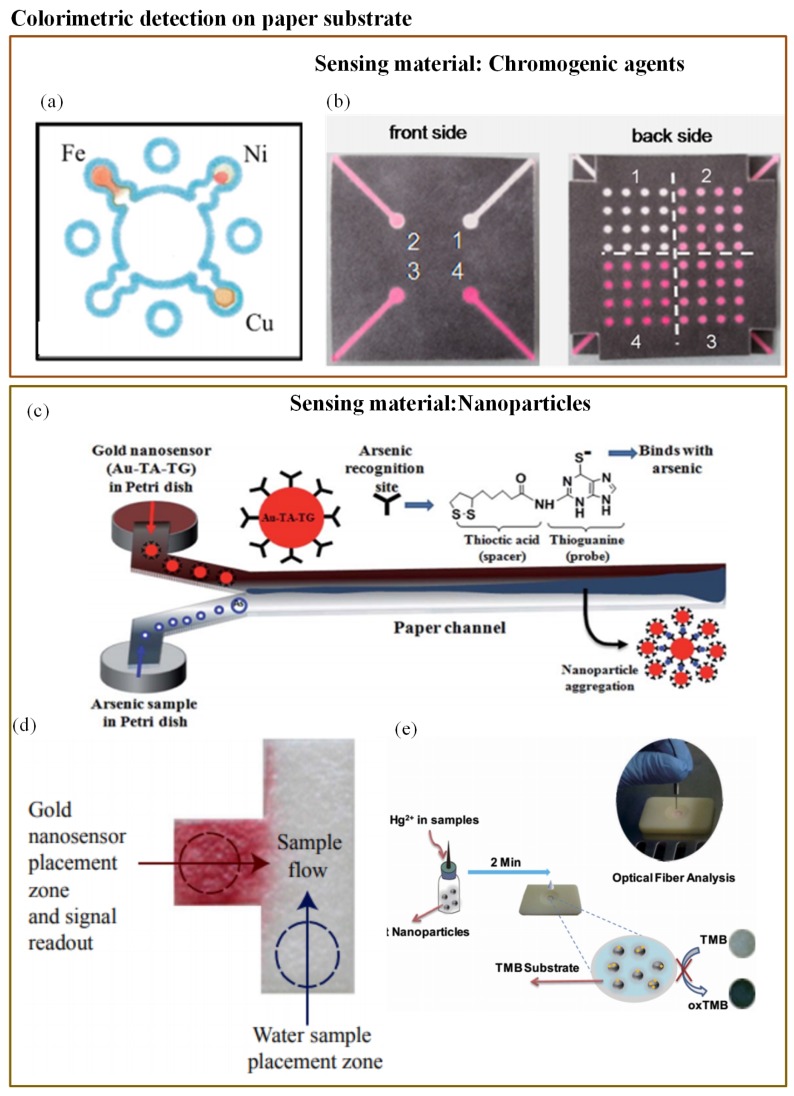
(**a**) Wax-printed μPADs for colorimetric detection of Fe, Cu, and Ni [172]; (**b**) 3-D paper microfluidics for metal ion detection [138]; (**c**) working principle of As(III) detector based on modified AuNP [141]; (**d**) T-shaped μPAD with functionalized AuNp for As(III) detection [142]; and (**e**) rapid detection of Pb^2+^ with MUA-modified AuNP [140].

Colorimetry is the most commonly used technique in microfluidics. The majority of the studied optical-based microfluidic devices used in heavy metal detection are paper-based colorimetric sensors. Colorimetric detection is performed in the dark, and so it is free from ambient light interference [171]. Mentele et al. [172] reported a paper-based colorimetric device (μPADs) for metal ion (Fe, Cu, and Ni) detection (Figure 7a). This method provides a rapid and an inexpensive way of metal ion detection. The possibility of utilizing paper microfluidics as a 3-D device was proved by Wang et al. (Figure 7b) [138]. They developed a 3-D paper-based microfluidic device for multiplex heavy metal (Cu (II), Ni (II), Cd (II), and Cr (VI)) detection through a simple combination of patterned paper by wax printing, tape, and stacking. The colorimetric determination was performed in association with a smartphone camera. The developed technique was rapid, low-cost, and user-friendly.

To avoid toxic chemical reactions in arsenic analysis, researchers have investigated and found gold nanoparticles (AuNPs) to be promising sensor materials in the colorimetric probe. Nath et al. [141] determined the use of As(III) with the help of simple paper-based microfluidics along with a gold nano-sensor (Au–TA–TG). The steady flow rate of the paper substrate pores allowed a very low concentration of arsenic to remain in a microchannel for a long enough period so that it interacted with the nano-sensor. A rapid reaction of Au–TA–TG with arsenic ions resulted in a visible dark bluish-black precipitate at the interfacial zone. The working principle is illustrated in Figure 7c. However, these μPADs were not tested against groundwater samples. When a similar implementation was tested with groundwater samples, the interference from several naturally occurring metals was observed [142]. To eliminate this limitation, Chowdury et al. [142] developed a T-shaped μPAD using the same functionalized gold nanoparticles (Au–TA–Au) as illustrated in Figure 7d. Additionally, they adjusted the pH value of the water sample to avoid other metal interferences. However, this assay provided just a qualitative result. Chen et al. [139] reported one more user-friendly and rapid μPAD for mercury(II) ion (Hg^2+^) measurement in water, for which they made use of oxidization of tetramethylbenzidine due to platinum nanoparticles and suppression of the reaction due to the presence of (Hg^2+^) ion. The whole interaction resulted in a visible colour change that was provided as a digital readout through the fiber optic module (Figure 7e). The sensor was capable of measuring (Hg^2+^) concentrations up until 0.01μM.

**Figure 8:**
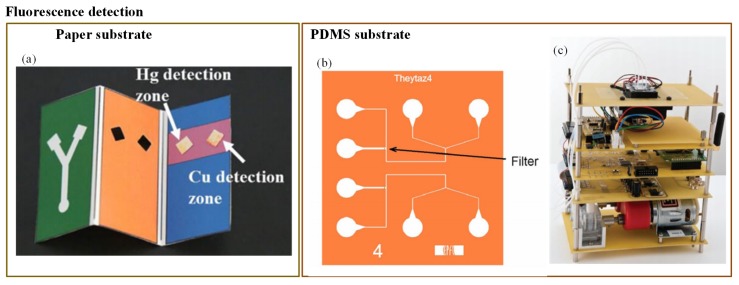
(**a**) Three-dimensional paper-based fluorescence detection of Cu^2+^ and Hg^2+^ [144]; (**b**) *E. coli*-based fluorescence detection of As(III) [146]; and (**c**) fluorescence detection of As(III) using portable bioreporter [145].

Fan et al. [140] designed a portable, power-free microfluidic device to detect lead (Pb^2+^). They detected Pb^2+^ with MUA-modified AuNPs (MUA-AuNPs). The chemical reaction between Pb^2+^ and MUA caused the aggregation of the modified nanoparticles, which in turn produced the solution colour change from red to purple. The output could be observed with the bare eye with the help of water drops. It was a rapid and inexpensive method with an LOD of 10 μM. In 2017, Bonyar et al. [143] developed a custom-tailored colorimetric semiautomated portable device for As(III) detection in drinking water. They integrated a commercially available arsenic test kit into a disposable microfluidic cartridge, as shown in Figure 8a. The Gutzeit reaction was carried out in the cartridge with automatic camera-based colour evaluation. The entire operation was easy to perform due to its user-friendly semiautomatic action and required approximately 1 h to obtain a result.

Miniaturization of fluorescence detection was possible due to the use of the light-emitting diodes (LEDs) in the optical detection system. LEDs can emit at various wavelengths, and they can easily fit into typical chip features [37]. Fluorescence detection is an extremely sensitive technique. However, according to Li et al. [37], its major limitation is that it can be used with the analytes that have native fluorescence or that can easily be fluorescently labelled. Still, many researchers have employed fluorescence detection to determine water pollutants. Qi et al. [144] developed a 3-D paper-based fluorescence sensor to determine Cu^2+^ and Hg^2+^ ions. It was based on a combination of quantum dots (QDs) and an ion imprinting technique on 3-D origami paper. CdTe QDs were implanted on the exterior of the glass fiber paper (Figure 8a). The change in fluorescence was produced due to the transfer of the photo luminescent energy of the QDs to its ion imprinting–QD complex. Bacterial bioassays have shown better performance in arsenic detection compared to a chemical field kit [173]. Theytaz et al. [146] created a microfluidic chip containing immobilized *E. coli* biosensor bacteria (Figure 8b). The *E. coli* generated green fluorescent protein in response to As(III). The major drawbacks of the developed method were its low LOD (50 μg/L) and the use of an epifluorescence microscope that made it a lab-based method. Similarly, Buffi et al. [174] demonstrated fluorescence detection of As(III) with the help of a bacteria-based bioassay.

The natural defence system of *E. coli* against As(III) was used to produce a fluorescence signal. The *E. coli* was embedded in small agarose beads. These beads were stored on a microfluidic chip; the fluorescence microscope was then used for signal detection. Hence, the bioassay cannot be considered as a portable device. Further, in 2014, this assay was enhanced by Truffer et al. [145]. They incorporated an electronic device with a small optical setup to measure fluorescence from bacterial reporter cells (Figure 8c). As a result, the device displayed significant potential for field measurements.

Currently, SERS integration with LoC devices is rapidly being adopted in biological and environmental analysis. Qi et al. [147] displayed prominent potential in integrating SERS technology with microfluidics in the field of water quality monitoring. They implemented a continuous flow detection of As(III) ions rapidly. Silver nanoparticles were modified with glutathione/4-mercaptopyridine (GSH/4-MPY). As(III) has a high affinity towards GSH. Hence, as As(III) came in contact with GSH/4-MPY, aggregation of nanoparticles occurred that produced a Raman signal. The developed assay was highly sensitive and reproducible with the LOD of 0.67 ppb.

Som-Aum et al. [148] developed a highly sensitive microfluidic sensor based on the CL method that could detect As(III) in water. In this method, sorption of a As(V) pre-concentration in the form of vanadomolybdoarsenate heteropoly acid (VMoAs-HPA) ion-paired with hexadecyltrimethylammonium bromide on the surface of polystyrene beads packed in the microfluidic tool was observed. The matrix effect was removed by adding 1×10^−8^ M ethylenediaminetetraacetic acid to all work solutions. Additionally, the interference from phosphate and chromate was eliminated by the synthesis of sorption pre-concentration. That also helped to enhance the sensitivity. The method obtained LOD of 8.9×10^−8^ M within 5 min.

### 4.2. Nutrients

**Figure 9:**
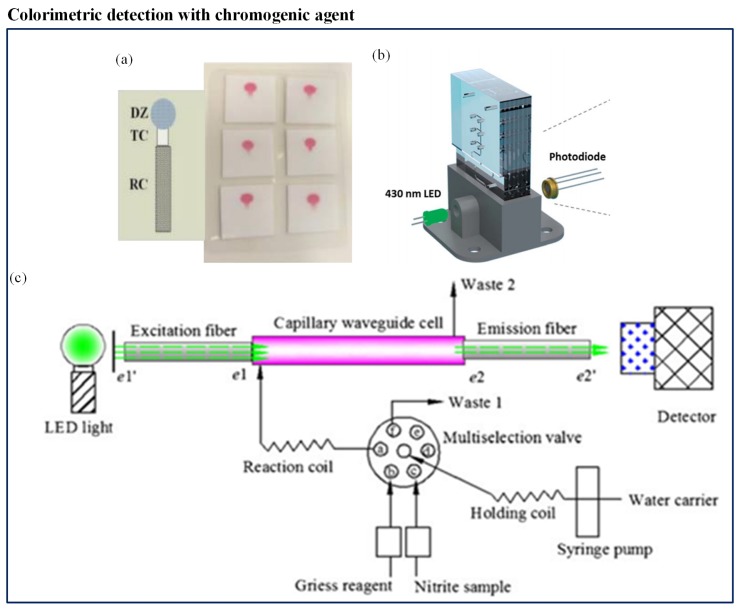
(**a**) Quantification of nitrite and nitrate using disposable μPAD [152]; (**b**) schematic of the flow system and detection cell of LED-based nitrate sensors [149]; and (**c**) schematic of the fiber-optic chemical sensor (FOCS) method for nitrite measurement [150].

Most of the available spectrophotometric methods for nitrate measurement in natural waters need conversion to the more reactive nitrite before detection. Different types of nitrate reduction methods have been presented, using a variety of reduction materials like hydrazine, copperized cadmium, zinc, nitrate reductase, and irradiation by ultraviolet light. Among all the methods available, the Griess assay is the most established method of colorimetric nitrite analysis [175]. Beaton et al. [153] first reported such a microfluidic-based colorimetric nitrate analysis using the Griess method. It was an in situ stand-alone system which was compact and consumed low power (1.5 W). Use of colored polymethylmethacrylate (PMMA) helped to reduce background light interference which made it a high-sensitivity system. The system displayed detection with high-resolution and produced a better output with detection limit of 0.02 μM for nitrite and 0.025 μM for nitrate. Another microfluidic method was developed by Khanfar et al. [151] to detect nitrate ions in water in an inexpensive and portable way. It was based on the Griess procedure. The microfluidic chip had a long-coated PMMA channel constructed with layers of different thicknesses. The detection system included an LED and photodiode. However, its LOD was low (0.0782 ppm). Jayawardane et al. [152] developed a cost-effective disposable μPAD to determine nitrite and nitrate (Figure 9a). This method also used a Griess reaction for nitrite determination. However, for nitrate detection, nitrate was reduced to nitrite using zinc microparticles inside the μPAD channel. The μPAD was fabricated by an inkjet printing method. The hydrophilic μPAD channel was integrated with zinc microparticles and worked as a virtual flow-through solid-phase reactor, which was a unique concept. The LODs of this method were 1.0 μM and 19 μM for nitrite and nitrate, respectively. This user-friendly method was suitable for a filed measurement. Recently, Vincent et al. [154] deployed a sensor within the Seaglider. The sensor employed colorimetric detection, using the Griess assay to determine nitrate and nitrite. The sensor was comprised of a three-layer PMMA chip. The chip included microchannels, mixers, photodiodes, and LED. The chip was installed with electronics, valves, and syringe pump. Eventually, the chip was covered in a housing that was filled with mineral oil and consisted of internally fitted pressure-compensating bladder. The LOD of the system was 20 nM. Cogan et al. [149] constructed a low-cost, robust microfluidic sensing platform and an LED-based optical detection system to determine nitrate in natural waters and wastewater. It was a complete system consisting of colorimetric measurement unit, a power unit, wireless communication, storage for sampling, reagent, and waste in a small unit. The chromotropic method for nitrate analysis was applied. The colorimetric measurement unit included a LED and a photodiode (Figure 9b). The author claimed advantages such as ease of operation, inexpensive, low consumption of power, high throughput, limited waste generation, and compactness in design. Xiong et al. [150] designed a novel miniaturized cost-effective colorimetric fiber-optic chemical sensor (FOCS) system for nitrite detection through interfacing with a microfluidic capillary waveguide. It was based on the Griess–Ilosvay reaction. When the reaction occurred between nitrite and Griess reagents, it generated colorimetric azo dye. The light intensity was changed when the light interacted with the azo dye. The method achieved LOD of 7 μg/L. The sensor comprised three sections: a capillary waveguide flow cell, a light source connected with an excitation fiber, and a detector connected with a detection fiber, as shown in Figure 9c. The microfluidic capillary waveguide also acted like a disposable sampling vessel, a reagent flow-through cell, and a light transmission element.

### 4.3. Pathogens

**Figure 10:**
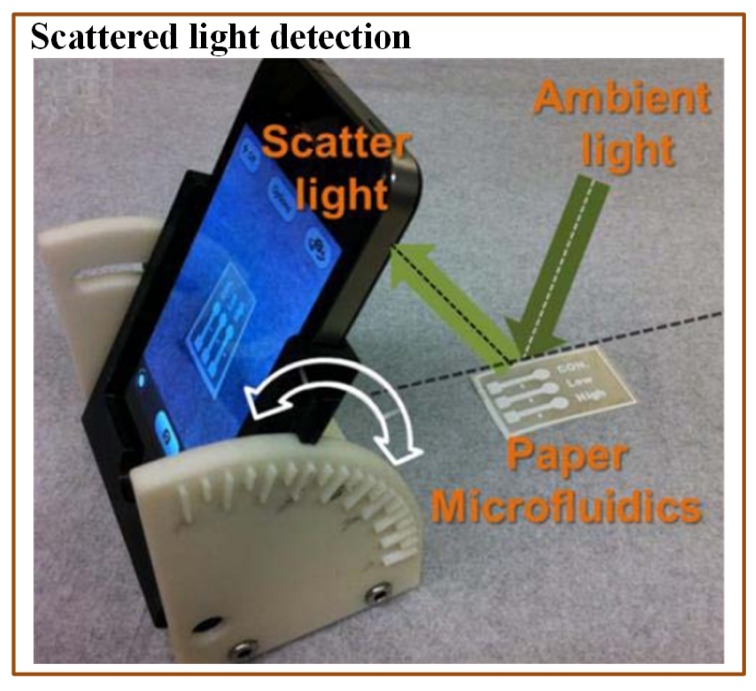
Scheme indicating a mobile-based multichannel paper chip for rapid *E. coli* detection [158].

A colorimetric method is commonly used in optical detection of various pathogens due to its simplicity and easy readouts. Many times, imaging devices (cell phone camera, portable scanner, and digital camera) are incorporated with the colorimetric methods to provide the analysis interpretation. Wang et al. [176] demonstrated a paper-based *E. coli* detection method. They used methylsilsesquioxane (MSQ) barriers to lyse the bacterial cells before the analysis. They also compared MSQ with other barrier materials, wax and alkylketene dimer (AKD). For this purpose, they printed circular barriers of MSQ, AKD, and wax. They found MSQ barriers better than the other materials. The change in colour was recorded with the help of the iPhone 4S camera. Although the assay was affordable and rapid, it was just a qualitative indicator. Boehle et al. [161] developed a cost-effective paper-based colorimetric method to detect antimicrobial-resistant bacteria. This method could identify the presence of b-lactamase-mediated resistance. An array of paper wells was used to optimize the reaction between b-lactamase and nitrocefin. The time required for the analysis was approximately 1 h with LOD of 10 mU/mL. San et al. [158] developed another paper-based method to detect *E. coli* from field water samples in association with a smartphone. The multichannel paper chip was preloaded with antibody-conjugated beads. The water sample was applied to the inlet of the paper chip, which allowed passing of the bacterial antigens. The smartphone was used to capture the digital images at some angle and to measure the light scatter intensity coming from microbead immunoagglutination (Figure 10). The entire analysis time was just 90 s. The assay was simple to use and did not require any external hardware. The only necessary device was a smartphone with a built-in gyro-sensor and an installed software application.

Fluorescence detection is another common method in optical pathogen detection. Golberg et al. [156] reported specific capture and detection of bacterial contamination in water. They developed a unit which consisted of *E. coli* seizing along with droplet microfluidics, portable proprietary fluorescence microscopy, and cloud-based data management and sharing. In this scheme, they used magnetic beads coupled with specific antibodies to capture *E. coli*. Further, the seized *E. coli* were conjugated fluorescently labeled antibodies. Subsequently, automated optical fluorescence microscopy was used for the purpose of detection. The entire water quality analysis took place within eight hours from sample collection to online result display. Malec et al. [157] proposed a labelled base biosensor where the *E. coli* was labelled with streptavidin-coated magnetic markers developing compounds. Video microscopy along with particle tracking software were utilised for quantitative measurement. The developed microfluidic platform was integrated with microconductors that generated a magnetic field gradient.

When the fluid with the magnetically labelled bacteria (MLB) was brought into the microfluidic platform, a magnetic field gradient accelerated the MLB towards the outlet. The method was able to provide a real-time approach for the detection of pathogens from a small-volume liquid sample.

Many researchers implemented integration of a polymerase chain reaction (PCR) test on a microfluidic platform. For example, Dharmasiri et al. [155] developed a PMMA microfluidic chip with eight parallel inputs covalently bonded with polyclonal antibodies. The chip was used for the isolation and detection of *E. coli*. The quantification was performed after isolation by an off-chip real-time quantitative PCR test. Fluorescent microscopy was used to examine the fluorescently labelled cells in the microfluidic chip’s channels. This entire process took just under five hours. The LOD was approximately 6 CFU. Li et al. [160] developed an integrated microfluidic device for rapid detection of pathogenic rotavirus. The device integrated reverse transcription (RT) and PCR with an online fluorescence detection technique. The microfluidic section incorporated the grooved copper heating block for RT and a heated cylinder for amplification. The RT-PCR technique with fluorescence microscopy was able to amplify and measure rotavirus RNA within one hour.

**Figure 11:**
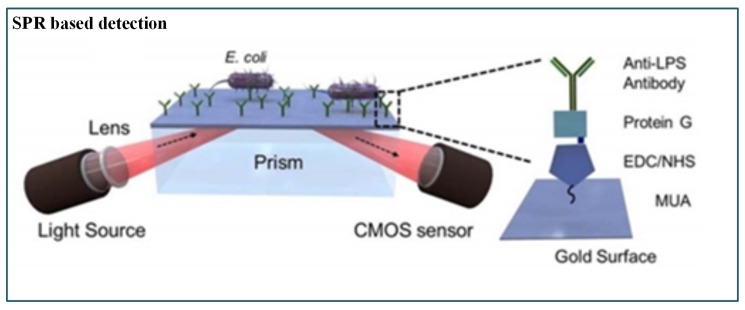
Schematic of SPR-based pathogenic detection [159].

Tokel et al. [159] presented a portable, multiplex, inexpensive microfluidic-integrated SPR platform for rapid detection of bacteria such as *E. coli* and *S. aureus*. It was a label-free pathogen detection platform consisting of microfluidic and SPR technologies. This method utilized a Protein G-based surface chemistry for *E. coli* determination that allowed immobilization of antibodies in a favourable orientation. However, the result was presented as a graph, whereas a direct readout could have been more appropriate (Figure 11).

## 5. Discussion and Outlook

**Figure 12:**
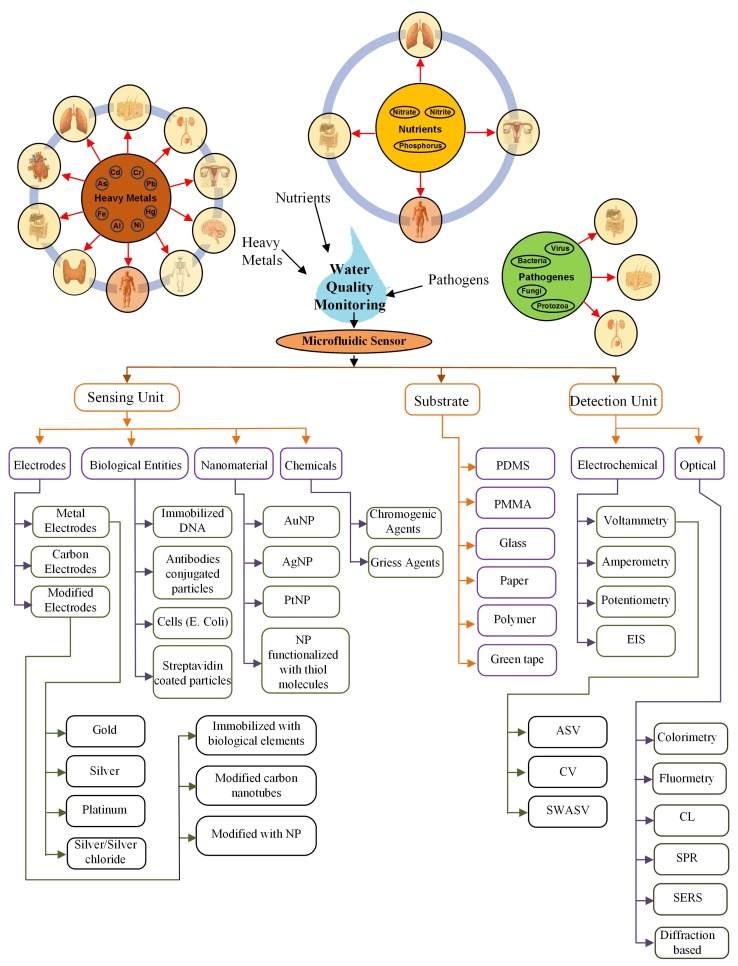
Summary.

Regular water quality monitoring is a “must” due to the harmful effects of water contaminants on the various functional systems of a human body. A microfluidic-based sensor is the most suitable method for this purpose. The sensor comprises of a sensing and detection unit on the microfluidic substrate. This review explores several sensors mainly with the sensing unit mostly based on chemicals, biological elements, electrodes, and nanomaterials. The materials used for the sensing unit and substrates are listed in Figure 12.

Furthermore, the review includes the sensors based on two signal transduction methods: electrochemical and optical detection.

Electrochemical detection is a big hope for microfluidic devices considering its high sensitivity, selectivity, miniaturization, and the possibility of mass production. Its adaptability with different microfabricated electronic parts leads to a portable device. To increase the sensitivity of electrochemical sensors, modification of electrodes with bioreporters or nanomaterials is advisable. However, a significant concern in regard to these sensors is the fabrication of chemically modified electrodes since it involves a very complicated process. Various optical sensing methods are successfully used in association with microfluidics including colorimetry, chemiluminescence, fluorescence, SPR, etc. The colorimetric analysis provides simple qualitative results in terms of a colour change. The colorimetric methods give relative results; they cannot yield exact quantitative results. These methods also require washing or rinsing steps before the next measurement can be taken in the microfluidic chip. Compared to colorimetric methods, chemiluminescence techniques have higher sensitivity. Also, the elimination of an external light source makes the instrumentation simple. However, the availability of a limited number of chemiluminescence reagents is the main disadvantage of this technique. The fluorescence detection is another highly sensitive method. Still, it is limited to analytes that possess inherent fluorescence or that can be labelled fluorescently. SPR and SERS are highly sensitive and selective optical detection methods. However, integration of these methods with a microfluidic platform can be an issue due to non-portable instrumentation. An optical diffraction method is yet another sensitive detection method, though it still remains unaddressed in water quality monitoring. This method can produce highly sensitive results and can be incorporated with microfluidic technology. Table 6 summarizes advantages and disadvantages of electrochemical and optical methods individually.

Field implementation is also an important aspect of discussion while discussing sensitivity and selectivity. Though there are a few challenges while implementing these sensors in the real world, the major hurdle associated with these sensors is field deployability. Many sensors discussed here represent the possibility of in situ and real-time measurement. However, those remain lab-based methods due to interfacing of lab-based measuring devices. Another challenge is in real sample measurement due to interference of the matrix effect. Usually, insoluble particles are suspended in the natural samples, which can influence the detection methods. In the case of optical detection, such particles can change the analyte concentration due to the stimulation of light scattering, while in electrochemical detection, such particles can modify the chemical electrodes. These challenges can be addressed by incorporating suitable measuring as well as filtering devices along with the sensing mechanism using microfluidics.

Microfluidic technology plays an important role in making the water quality sensors field effective, as size reduction and automation are highly possible through this technology. However, the technology has its own challenges such as improper mixing in microchannels caused by laminar flows through it, which can potentially be addressed by implementing passive mixing microfluidic structures. Another challenge is the fabrication of microchannels with random geometries. Additionally, fabrication of the microfluidic sensor may sometimes remain a laboratory prototype that needs access to clean room equipment and trained staff to operate. It is possible to overcome these challenges with the help of rapidly emerging 3-D printing technology.

## 6. Conclusions

In the present review, microfluidic-based sensors for water quality monitoring have been extensively discussed in detail. This includes a comparison of microfluidic-based electrochemical and optical methods with advantages and disadvantages for the detection of contaminants such as heavy metals, nutrients, and pathogens in water that have been published in the last decade. Water quality analysis with microfluidics is a flourishing technology as it contributes to rapid, economical, and user-friendly detection methods. It is especially suitable for in situ testing, particularly in limited-resource circumstances. The current challenges with in situ testing and real samples are also discussed in the review. Such challenges can be addressed by including 3-D printing technology along with the microfluidic platform.

## Figures and Tables

**Table 1:** Contaminants and their potential health effects.

Contaminant MCL (mg/L)	Cancer	Developmental/ Reproductive	Neurologic	Other	Sources	Ref.
Arsenic (0.01)	Skin, internal	SAB	Peripheral	Cardiovascular, immunologic, dermatologic	Geothermal activity, agricultural application, mining and smelting, industrial applications, industrial and electronics wastes	[54,55,56,57,58,59]
Lead (0.01)	Internal (OCC)	Birth defects	Autism, dyslexia, hyperactivity	Haemoprotein, weight loss, muscular weakness, paralysis, kidney damage	Natural deposits, mining, manufacturing process, and fossil fuel burning	[58,60,61]
Mercury (0.002)	Internal	Damage to fetus	Neurobehavioral disorders	Cardiovascular, thyroid, asthma, nausea and vomiting, diarrhea, skin rashes, cardiovascular	Natural deposits, land runoff agricultural and industrial applications, paper and pulp preservatives	[58,62,63,64]
Cadmium (0.004)	Pancreatic ovarian breast	Preterm birth, LBW	Neuron cell death	Leading to kidney disease, oxidative stress, osteoporosis, DNA damage	Natural deposits, mining, smelting, tobacco smoking, disposal of sewage	[58,65,66,67]
Chromium (0.05)	Lung and gastrointestinal	NA	NA	Nausea and vomiting, low blood sugar, damage to liver and kidney, dermatological	Natural deposits in soil and rocks, volcano irruption, coal and oil combustion, sewage sludge, cement production	[47,58,68,69]
Nickel (0.02)	Lung and nasal	NA	NA	Lung disease, skin diseases, liver toxicity	Volcanic eruption, forest fires, industrial and domestic wastewater, sewage sludge	[70,71]
Aluminum (0.05 to 0.2)	NA	NA	NA	Nausea and vomiting, mouth ulcers, diarrhea, skin rashes, arthritic pain	Industrial applications	[58,72,73]
Iron (0.3)	Lung	NA	NA	Gastrointestinal bleeding, vomiting and diarrhea	Natural deposits, corroded iron pipes	[58]
Nitrate (50)	Internal	SAB blue baby syndrome	NA	Gastric problems, Parkinson’s disease	Natural deposits, agricultural usage, animal waste, septic tanks, sewage sludge	[74,75]
Nitrite (0.2)		Blue baby syndrome		Gastric problems	Natural deposits, agricultural usage, animal waste, septic tanks	[76,77,78,79]
Pesticide- 1,3-dichloropropene (0.02)	Carcinogenic	LBW	NA tumors	Skin irritations	Agricultural applications	[74]
*E. coli* (less than 1/100 mL)	NA	NA	NA	Kidney failure, anemia, diarrhea, and other serious health problems	sewage leakage, animal waste	[80,81]
Rotavirus Zero	NA	NA	NA	Vomiting, dehydration, severe fatigue	disposal of untreated wastewater	[46]
Protozoa (Less than 1(oo) cyst/100L)	NA	NA	NA	Diarrhea, fatigue, nausea, abdominal cramps	Faecal contamination	[82]

NA: Not Applicable; LBW: Low birth weight; MCL: Maximum contamination level; OCC: Occult cancer; SAB: Spontaneous abortion.

**Table 2:** Comparison of electrochemical methods.

Electrochemical Methods							
Target Analyte	Detection Principle	Sensing Element	Sensing Material	Substrate	LOD	Real Sample	Ref.
Hg^+2^	ASV	Electrode system	WE: Au	PDMS	3 ppb	No	[101]
			CE: Au				
			RE: Ag				
Pb^+2^ and Hg^+2^	CV	Electrode system	WE: carbon	Polyethylene terephthalate	50 μM each	No	[104]
			CE: Ag				
			RE: Ag/Cl				
Pb^+2^	SWASV	Electrode system	WE: Ag	Polymer	0.55 ppb	No	[99]
			CE/QRE: Ag				
Pb^+2^ and Hg^+2^	SWASV	Electrode system	WE: carbon	Paper	2.0 and 2.3 ppb, resp. water	Soda water and dirty ground	[111]
			CE: carbon				
			RE: Ag pseudo				
As	CV	Electrode system	WE: Ag	Plastic	1 ppb	No	[102]
			CE: Carbon				
			RE: Ag/AgCl				
As	SWASV	Electrode system	WE: Au/SWCNT	PDMS	4.5 ppb	No	[103]
			CE and RE: SWCNT				
As(III)	Amperometry	Bioreporter with electrode system	WE: Au	Plastic	0.8 ppb	Tap and ground water	[112]
			CE and RE: Ag and *E. coli*				
Nitrate	CV	Electrode system	WE and RE: Ag	Glass	25 ppb		[113]
			CE: Au				
Nitrate	CV	Electrode system	WE: Ag	Glass	0.2 ppm	Field and environmental water	[114]
			CE: Au				
			RE: Ag				
Nitrate and Potassium	Potentiometric	Electrode system	WE: Polymeric membrane	Green tapes	9.56 and 0.81 mg/L, respectively	Water from recycling unit	[115]
			RE: Ag/AgCl				
Nitrate	Potentiometric	Electrode system with modified working electrode	WE: f-MWCNTs RE:Ag/AgCl	Lipophilic carbon nanotubes	5×10^−7^ M	Desalinated seawater	[116]
Nitrate	EIS	Electrode system with modified working electrode	WE: NiR/nTiO2-GF CE: Au RE: Ag/AgCl	PDMS	1 μM	No	[117]
Nitrate and Nitrite	SWV	Electrode system with modified working electrode	WE: Cu/MWCNT/RGO/GCE CE: Pt-wire RE: Ag/AgCl	GCE	20 and 30 nM, resp.	Tap and mineral water	[77]
*E. coli*	Voltammetry	Electrode system with modified working electrode	WE: immobilized DNA probe on Au CE and RE: Pt	Glass	100 nM	No	[118]
Hepatitis B	ASV	Electrode system	WE: GCE CE: Pt wire RE: Ag/AgCl and DNA modified AgNP	Paper	85 pM	No	[119]
*E. coli*	Amperometry	Immunoassays	Antibody	PMMA	50 CFU/mL	Real sample	[120]
*E.coli*	Positive dielectrophoresis	Sensing and focusing electrode	Not specified	PDMS	300 CFU/mL	No	[121]
*E. coli*	EIS	Interdigitated electrodes	Modified silicon sensor chip	Silicon and PDMS	10 cells/mL	No	[122]
*E. coli* and *S. aureus*	EIS	Coplanar electrode	Au electrode	Silicon	522 cells/mL	No	[123]
*E. coli* and Enterococci	Coulter principle	Microfluidic sensing chip	Resistance detection circuit	PDMS	Individual cell	Ballast water sample	[124]
Salmonella typhimurium	Impedance analyzer	Interdigitated electrodes	Au electrode with immobilized antibodies	PDMS	3×10^3^ CFU/mL	No	[125]

LOD: Limit of detection; ASV: Anodic stripping voltammetry; PDMS: Polydimethylsiloxane; PMMA: Poly(methyl methacrylate); CV: Cyclic voltammetry; SWASV: Square-wave anodic stripping voltammetry; QRE: Quasi-reference electrode; WE:Working electrode; CE: Counter electrode; RE: Reference electrode; EIS: Electrochemical impedance spectroscopy; SWV: Square-wave voltammetry; RGO: Reduced graphene oxide; GCE: Glassy carbon electrode; -MWCNTs: Functionalised- multiwall carbon nanotube.

**Table 3:** Commercially available electrochemical sensors.

Target Analyte	Measurement Principle	Measuring Range	Features	Company	Ref.
Arsenic	Paper-based electrochemistry test strips	Not specified	Easy-to-use, quantitative, fast, low cost, nontoxic, disposable	Bio Nano Consulting	[126]
Copper, lead, and cadmium	Stripping square wave voltammetry with carbon–carbon–silver electrodes	Not specified	Easy-to-use, quantitative, simple, easy to use, cost-effective	PalmSense	[127]
Heavy metals	Potentiometric cell with carbon–bismuth electrodes	Not specified	Simultaneous analysis, portable systems, in situ results, low cost	GTQ (Chemical transducers research Group)	[128]
Nitrate	Potentiometric cell with liquid membrane ion selective electrodes	0.6 to 200.0 ppm	Detection of nitrate–nitrogen in freshwater samples	MEDIRAY	[129]
Nitrate	Potentiometric cell with ion-selective electrodes	0.5 to 450.0 mg/L	Simple to use, callibration-free operation	Xylem	[130]
Nitrate	Potentiometric cell with ion-selective electrodes	1 to 14,000 mg/L	Easy to use, portable	Vernier	[131]
Nitrate	Potentiometric cell with ion-selective electrodes	0.62 to 6200 ppm	Replaceable sensing modules, durable polyetherimide (PEI) body, BNC(Bayonet Neill–Concelman) connection	HANNA instruments	[132]

**Table 4:** Comparison of optical methods.

Optical Methods							
Target Analyte	Detection Principle	Sensing Element	Sensing Material	Substrate	LOD	Real Sample	Ref.
Cu(II) Ni(II) Cd(II) Cr(VI)	Colorimetric	Chemical compound	Sodium diethyldithiocarbamate Dimethylglyoxime Cadion Diphenylcarbazide	Paper	0.29 ppm 0.33 ppm 0.19 ppm 0.35 ppm	Distilled water Reservoir water Beach water	[138]
Hg^2+^	Colorimetric	Nanoparticles	Platinum nanoparticles and 3,3,5,5-tetramethylbenzidine	Paper	0.01 uM	Pond and tap water	[139]
Pb^2+^	Colorimetric	Functionalized nanoparticles	AuNP functionalized with 11-mercaptoundecanoic acid	PDMS	10 μM	No	[140]
As(III)	Colorimetric	Functionalized nanoparticles	AuNP functionalized with α-lipoic acid and thioguanine	Paper	1.0 ppb	No	[141]
As	Colorimetric	Functionalized nanoparticles	AuNP functionalized with α-lipoic acid	Paper	Quality analysis	Bangladesh groundwater	[142]
As(III)	Colorimetric	Hach®EZ Arsenic Test Kit	Standard Gutzeit reaction reagents	Plastic	3 μg/L	No	[143]
Cu^2+^ and Hg^2+^	Fluorescence	Quantum dots	CdTe quantum dots	Paper	0.035 μg/L 0.056 μg/L	Lake and sea water	[144]
As(III)	Fluorescence	Bioreporter cell	*E. coli*	PDMS	10 μg/L	Tap water	[145]
As(III)	Fluorescence	Bioreporter cell	*E. coli*	PDMS	50 μg/L	No	[146]
As(III)	SERS	Functionalized nanoparticles	AgNP functionalized with glutathione/4-mercaptopyridine	PDMS	0.67 ppb	Tap water Mineral water	[147]
As(IV)	CL	Chemical compound	Luminol and Vanadomolybdoarsenate heteropoly acid	PDMS	8.9×10^−8^ M	Tap water	[148]
Nitrate	Colorimetric	Chromogenic agent	Chromotropic acid and Sulphuric acid	PDMS	0.70 mg/L	Drinking water, freshwater, wastewater, and sea water	[149]
Nitrate	Colorimetric	Chromogenic agent	Griess reagent	Fiber	7 μg/L	Lake water Tap water	[150]
Nitrate	Colorimetric	Chromogenic agent	Griess reagent	PMMA	0.0782 ppm	Tap water Bottled drinking water Home-filtered water	[151]
Nitrite and Nitrate	Colorimetric	Chromogenic agent	Griess reagent Zinc microparticles	Paper	1.0 μM 19 μM	Tap water and synthetic water	[152]
Nitrite and Nitrate	Colorimetric	Chromogenic agent	Griess reagent Imidazole buffer	PMMA	0.02 μM 0.025 μM	River water	[153]
Nitrite and Nitrate	Colorimetric	Chromogenic agent	Griess reagent Copper-activated cadmium column	PMMA	20 nM	Sea water	[154]
*E. coli*	PCR	Biological elements	Polyclonal antibodies	PMMA	6 CFU	Recreational lake water, waste water	[155]
*E. coli*	Fluorescence	Biological elements	Magnetic beads conjugated with antibodies	PDMS	??	Drinking water	[156]
*E. coli*	Fluorescence	Biological elements	Streptavidin-coated magnetic markers	PDMS	??		[157]
*E. coli*	Light scattering	Biological elements	Antibody-conjugated beads	Paper	10 CFU/mL	Field water	[158]
*E. coli* *S. aureus*	SPR	Biological elements	Au surface modified with MUA, EDC/NHS, Protein G and anti-LPS antibody	PMMA	??	No	[159]
Roravirus	Fluorescence		Graphene oxide	Glass	10^5^ PFU/mL	No	[160]
Antimicrobial- resistant bacteria	Colorimetric	Chromogenic agent	Nitrocefin	Paper	10 mU/mL	Sewage water, river water	[161]

CL: Chemiluminescence; ??: Not specified; SPR: Surface plasmon resonance; MUA: 11-mercaptoundecanoic acid; LPS: Lipopolysaccharide; EDC/NHS: Ethyl-3-(3-dimethy-laminopropyl) carbodiimide hydrochloride/N-Hydrosuccinimide.

**Table 5:** Commercially available optical sensors.

Target Analyte	Measurement Principle	Measuring Range	Features	Company	Ref.
Arsenic	Kit-based colorimetric	0 to 500 ppb	Easy-to-use, effective way	Hach	[162]
Arsenic	Kit-based colorimetric	0 to 500 ppb	Result in 12 min, 100 tests per kit	FilterWater.com	[163]
Arsenic	Kit-based digital colorimetric	2 to 100 ppb	Reaction time 20 mins	Palintest Water analysis technology	[164]
Arsenic	Atomic fluorescence spectrometry	10 ppt	Easy-to-learn and easy-to-use system, can be automated	P S Analytical	[165]
Lead, thallium, mercury, cadmium iron, nickel, and zinc	Color-based visual detection	Not specified	Simple to use, results in 15 to 60 s, low-cost analysis	ChemSee	[166]
Nitrate	Portable photometer	0.0 to 30.0 ppm	Easy to use, not suitable for seawater	HANNA instruments	[167]
Nitrate	UV absorbance with	0 to 50 mg/L	Modern communication systems allow data to be accessed in real-time	HydroMetrics	[168]
Nitrate	UV absorbance with	0.05 to 200 mg/L	Access with web browser, optional anti-fouling wiper, flexible sensor options	OTT ecoN	[169]
*C. jejuni, C. coli,* *C. upsaliensis*, and *C. lari*	PCR-campylobacter detection kit	Not specified	Specific, rapid, and reliable detection; amplification limit of one copy per reaction; ready-to-use kit	BioVision	[170]

**Table 6:** Advantages and disadvantages of detection methods.

Method	Advantages	Disadvantages
Electrochemical	High selectivity High selectivity Miniaturized electrodes makes the system portable Possibility of mass production	Tedious fabrication process of electrode
Colorimetric	Simple analysis Provides qualitative results Quick response	Provides relative output
CL	High sensitivity Does not require external light source Portable	Limited number of CL reagents available
Fluorescence	High sensitivity High selsectivity Portable	Limited to analytes that possess inherent fluorescence External light source necessary
SPR	High sensitivity High selsectivity Label-free detection	Portability may be an issue
SERS	High sensitivity High selsectivity	Highly sensitive to environmental changes
Optical diffraction	High sensitivity High selsectivity ortable	Occasionally, signal enhancement by sequential amplification is necessary

## Abbreviations

The following abbreviations are used in this manuscript:

AKD	alkylketene dimer
ASV	anodic stripping voltammetry
AuNP	gold nanoparticles
BNC	Bayonet Neill–Concelman
CFU	colony forming units
CL	chemiluminescence
CV	cyclic voltammetry
*E. coli*	*Escherichia coli*
EDC	ethyl-3-(3-dimethy-laminopropyl) carbodiimide hydrochloride
EIP	electrochemical impedance spectroscopy
EIS	electrochemical impedance spectroscopy
f-MWCNT	functionalised- multiwall carbon nanotube
FOCS	fiber-optic chemical sensor
GCE	glassy carbon electrode
GF	graphene foam
LCR	inductance, capacitance, resistance
LEDs	light-emitting diodes
LoC	Lab-on-a-chip
LOD	limit of detection
LPS	lipopolysaccharide
LTTC	low-temperature co-fired ceramics
MB	methylene blue
MCL	maximum contamination level
MLB	magnetically labelled bacteria
MSQ	methylsilsesquioxane
MUA	11-mercaptoundecanoic acid
MWCNTs	multiwall carbon nanotubes
NHS	n-hydrosuccinimide
NiR	nitrate reductase
OCC	occult cancer
PDMS	polydimethylsiloxane
PMMA	polymethylmethacrylate
pDEP	positive dielectrophoretic
POC	point-of-care
QRE	quasi-reference electrode
RGO	reduced graphene oxide
SAM	self-assembled monolayer
SERS	surface-enhanced Raman scattering
SPR	surface plasmon resonance
*S. aureus*	*Staphylococcus aureus*
SWASV	square-wave anodic stripping voltammetry
SWCNTs	single-walled carbon nanotubes
SWV	square-wave voltammetry
μPADs	paper-based analytical devices
μPED	microfluidic paper-based electrochemical sensing device
